# 1338. Before and After: The Impact of the COVID-19 Pandemic on Influenza-Like Illness Trends in PAIVED

**DOI:** 10.1093/ofid/ofab466.1530

**Published:** 2021-12-04

**Authors:** Rhonda E Colombo, Stephanie A Richard, Christina Schofield, Limone Collins, Anuradha Ganesan, Casey Geaney, David Hrncir, Tahaniyat Lalani, Ana E Markelz, Ryan C Maves, Ryan C Maves, Bruce McClenathan, Katrin Mende, Jitu Modi, Jay R Montgomery, Srihari Seshadri, Catherine Skerrett, Christina Spooner, Gregory Utz, Alan Williams, Timothy Burgess, Christian L Coles

**Affiliations:** 1 Madigan Army Medical Center, Tacoma, WA, Infectious Disease Clinical Research Program, Bethesda, MD, and Henry M. Jackson Foundation for the Advancement of Military Medicine, Inc., Bethesda, MD, Tacoma, Washington; 2 Infectious Disease Clinical Research Program, Department of Preventive Medicine and Biostatistics, Uniformed Services University of the Health Sciences, Bethesda, MD and Henry M. Jackson Foundation, Bethesda, MD, Bethesda, Maryland; 3 Madigan Army Medical Center, Tacoma, WA, Tacoma, Washington; 4 Immunization Health Branch, Defense Health Agency Bethesda, MD, Falls Church, VA, San Diego, CA, Falls Church, VA; 5 Infectious Disease Clinical Research Program and the Henry M. Jackson Foundation for the Advancement of Military Medicine and Walter Reed National Military Medical Center, Bethesda, MD; 6 Walter Reed National Military Medical Center, Bethesda, MD; 7 Lackland Air Force Base & Carl R. Darnall Army Medical Center, San Antonio, Texas; 8 IDCRP, HJF, and NMCP, Bethesda, Maryland; 9 Brooke Army Medical Center, Fort Sam Houston, Texas; 10 Naval Medical Center San Diego, San Diego, CA and Infectious Disease Clinical Research Program, Bethesda, MD, San DIego, California; 11 Womack Army Medical Center, Fort Bragg, NC 28310, Fort Bragg, North Carolina; 12 Infectious Disease Clinical Research Program, Bethesda, MD, The Henry M. Jackson Foundation, Bethesda, MD, and Brooke Army Medical Center, Fort Sam Houston, TX, San Antonio, TX; 13 Naval Health Clinic Annapolis, Laurel, Maryland; 14 Defense Health Agency, Vienna, Virginia; 15 Immunization Health Branch, Defense Health Agency, Falls Church, VA; 16 Lackland Air Force Base, San Antonio, Texas; 17 Uniformed Services University of the Health Sciences, Bethesda, Maryland; 18 USUHS/FAM, Bethesda, Maryland; 19 Infectious Disease Clinical Research Program, Bethesda, Maryland; 20 Infectious Disease Clinical Research Program, Bethesda, MD, The Henry M. Jackson Foundation, Bethesda, MD, Bethesda, MD

## Abstract

**Background:**

The Pragmatic Assessment of Influenza Vaccine Effectiveness in the DoD (PAIVED) is a multicenter study assessing influenza vaccine effectiveness in active duty service members, retirees, and dependents. PAIVED recently completed its third year and offers a unique opportunity to examine influenza-like illness (ILI) trends prior to and during the COVID-19 pandemic in a prospective, well-defined cohort.

**Methods:**

During the 2018-19, 2019-20, and 2020-21 influenza seasons, PAIVED enrolled DoD beneficiaries presenting for annual influenza vaccination. After collecting baseline demographic data, participants were randomized to receive egg-based, cell-based, or recombinant-derived influenza vaccine. Weekly throughout the influenza season of enrollment, participants were surveyed electronically for ILI, defined as (1) having cough or sore throat, plus (2) feeling feverish/having chills or having body aches/fatigue. Participants with ILI completed a daily symptom diary for seven days and submitted a nasal swab for pathogen detection.

**Results:**

Over the three seasons, there were 10,656 PAIVED participants: 1514 (14.2%) in 2018-19, 5876 (55.1%) in 2019-20, and 3266 (30.6%) in 2020-21. The majority were male (68-73% per year) with a mean age of 34±14.8 years at enrollment. 2266 participants reported a total of 2673 unique ILIs. The highest percentage of participants with ILI was in 2019-20 (28.2%), versus 19.6% in 2018-19 and 9.6% in 2020-21. Figure 1 depicts the percent of individuals reporting ILI by week of the season for each of the PAIVED seasons. Notably, after March 21, 2020, the weekly incidence of participants reporting ILI never exceeded 1%.

Figure 1. Percent of PAIVED participants reporting ILI by week of season.

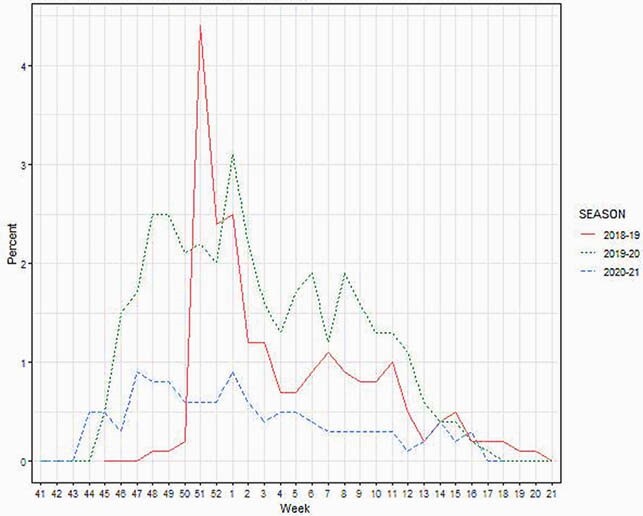

**Conclusion:**

The low incidence of reported ILI in PAIVED participants during the COVID-19 pandemic is consistent with national influenza surveillance reports of influenza and outpatient ILI activity, suggesting that mitigation measures taken to reduce transmission of SARS-CoV-2 reduced the spread of other respiratory viruses.

**Disclaimer:**

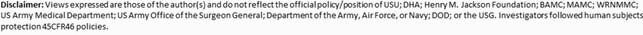

**Disclosures:**

**Ryan C. Maves, MD**, **EMD Serono** (Advisor or Review Panel member)**Heron Therapeutics** (Advisor or Review Panel member) **Jitu Modi, MD**, **GSK** (Speaker’s Bureau)

